# Identification and examination of nitrogen metabolic genes in *Lelliottia amnigena* PTJIIT1005 for their ability to perform nitrate remediation

**DOI:** 10.1186/s12864-023-09207-6

**Published:** 2023-03-09

**Authors:** Preeti Thakur, Pammi Gauba

**Affiliations:** 1 Department of Biotechnology, Jaypee Institute of Information & Technology, Noida, 201307 India; 2 Dean & Head of Biotechnology Department, Jaypee Institute of Information & Technology, Noida, Uttar Pradesh 201307 India

**Keywords:** *Lelliottia amnigena*, N-metabolic pathway, Genome sequence, Assimilation, Denitrification

## Abstract

*Lelliottia amnigena* PTJIIT1005 is a bacterium that utilizes nitrate as the sole nitrogen source and can remediate nitrate from media**.** The annotation was done related to nitrogen metabolic genes using the PATRIC, RAST tools, and PGAP from the genome sequence of this bacterium. Multiple sequence alignments and phylogenetic analysis of respiratory nitrate reductase, assimilatory nitrate reductase, nitrite reductase, glutamine synthetase, hydroxylamine reductase, nitric oxide reductase genes from PTJIIT1005 were done to find out sequence identities with the most similar species. The identification of operon arrangement in bacteria was also identified. The PATRIC KEGG feature mapped the N-metabolic pathway to identify the chemical process, and the 3D structure of representative enzymes was also elucidated. The putative protein 3D structure was analyzed using I-TASSER software. It gave good quality protein models of all nitrogen metabolism genes and showed good sequence identity with reference templates, approximately 81–99%, except for two genes; assimilatory nitrate reductase and nitrite reductase. This study suggested that PTJIIT1005 can remove N-nitrate from water because of having N-assimilation and denitrification genes.

**Supplementary Information:**

The online version contains supplementary material available at 10.1186/s12864-023-09207-6.

## Introduction

*Lelliottia amnigena* PTJIIT1005 (formerly referred to as *Enterobacter amnigenus*) belongs to the family of Enterobacteriaceae, which is Gram-negative. This bacteria is found in soil, water, and sewage [1], utilizing nitrate for its growth. Genes associated with nitrate assimilation have contributed to this process. The genes which involved in nitrate assimilation are glutamine synthetase, nitrate, and nitrite reductase. These genes can convert nitrate (NO_3_^−^) to nitrite (NO_2_^−^), then nitrite into ammonium (NH_4_^+^), followed by ammonium into glutamine. Further, glutamine is assimilated by *L. amnigena* to increase its growth, and this nitrate assimilation step has been reported in algae, higher plants, yeast, and bacteria [2]. The final product of N-assimilation, i.e., ammonium, is integrated into carbon skeletons by glutamine synthetase-glutamate synthase pathway or glutamate dehydrogenase [3].

Nitrate reduction plays an essential role in N-cycle. Nitrate is reduced in nature in three ways: (i) utilizing nitrate as a source of nitrogen called nitrate assimilation (ii) nitrate as terminal electron acceptor and producing metabolic energy called nitrate respiration (iii) reduction of nitrate into ammonium called nitrate dissimilation. These are three different kinds of nitrate-reducing systems that have been reported in microbes [4–6]. Assimilatory nitrate reductase (Nas) is a cytoplasmic enzyme induced by nitrate, nitrite, NADH, or ferredoxin as a reductant. Dissimilatory nitrate reductase (Nar) is a membrane-bound enzyme associated with denitrification and anaerobic nitrate respiration. Eventually, dissimilatory periplasmic reductase (Nap) is involved in aerobic and anaerobic denitrification.

Nas has been grouped into three sub-groups based on the number of cofactors of electron transfer bound by the catalytic bis-MGD (Molybdopterin-guanine dinucleotide) subunit and characters of the probable electron donors to every enzyme. These enzymes are represented by the microbes *Synechococcus* sp. PCC7942, *Klebsiella oxytoca* and *Bacillus subtilis* [5]. For instance, *B. subtitlis* includes a nasABCDEF gene cluster within which nasB/C and nasD/E genes encode for assimilatory nitrate and nitrite reductase, respectively.

Bacteria can respire nitrate, dissimilating the produced nitrite to gaseous compounds or ammonia, called denitrification or dissimilatory nitrate reduction. Denitrification is a biological process that removes high nitrate from surface water and wastewater. Nitrate accumulates because of extensive usage of agricultural N-fertilizers [7]. Membrane-bound (Nar) and periplasmic nitrate reductase (Nap) are found among bacteria [8]. NarGHI and NarZYV are primary membrane-bound nitrate reductase that has been known in *E. coli* [9]. Besides, dissimilative nitrate reductase is also found in the periplasm, and this nitrate reductase is termed Nap. These are found in some members of Enterobacteriaceae like *Pseudomonas aeruginosa* [10], *E. coli* [11], and non-sulfur photosynthetic microbes like *Rhodobacter capsulatus* [12], *Paracoccus denitrificans* [13] and other microbes like *Desulfovibrio desulfuricans* [14], *Campylobacter jejuni* [15] and *Epsilonproteobacteria* [16].

This paper explored the whole genome sequence of our latest isolated strain, *Lelliottia amnigena* PTJIIT1005, from the polluted site of Yamuna River, Delhi, India (28.565^0^N 77.303°E). It can use nitrate as a nitrogen source and remediate nitrate from water. The genome sequence was determined to analyze nitrate assimilated and dissimilated related genes.

## Materials and methods

### Bacterial strain and growth conditions

The bacterial strain *Lelliottia amnigena* (designated as PTJIIT1005) was isolated and identified from a water sample from the Yamuna River in Delhi, India. A nutrient broth (pH -8.0) containing 1500 mg/l nitrate and artificial groundwater medium contains KH_2_PO_4_–0.235 g/l, K_2_HPO_4_–0.09 g/l, NH_4_Cl - 0.1 g/l, MgCl_2_–0.0248 g/l, CaCl_2_–0.141 g/l and 5 ml/l Vitamin and minerals mixture amended with acetate (10 mM final concentration) and KNO_3_^−^ was used for bacterial growth.

### Genome sequencing and genome annotation

Genomic DNA was extracted from bacteria using a Nucleospin Microbial DNA kit (Thermo Fischer Scientific) as per manufacturer instructions. Further, the QIASeq FX DNA Library Kit (Qiagen) prepared genomic libraries for sequencing. *L. amnigena* PTJIIT1005 genome was sequenced on NGS (Next Generation Sequencing) Illumina NovaSeq6000 Platform by Redcliffe Lifetech, Noida. A total of 9,365,132 raw reads were obtained; 8,596,940 Illumina reads were de novo assembled using Unicycler (version 0.4.4). The assembled genome sequence was annotated by the tool Prokka 1.12. The complete genome sequence was submitted to NCBI.

Average Nucleotide Identity (ANI) [17] measures nucleotide-level genome similarity between the coding regions of two genomes. The complete genome sequence was submitted in FASTA format as an input file. This tool gives the similarity index percentage [18]. ANI is computed using the formula [19]:

$$\underset{\ \textbf{G1}{\to}\textbf{G2}}{\textbf{gANI}}=\frac{{\underset{\textrm{bbh}}{\Sigma\ }}\left(\textrm{Percent}\ \textrm{Identity}{}_{.}{}^{\ast}\textrm{A}\textrm{lignment}\ \textrm{length}\right)}{\textrm{lengths}\ \textrm{of}\ \textrm{BBH}\ \textrm{genes}}$$

Genome annotation of *Lelliottia amnigena* was done by RAST (Rapid Annotation using Subsystems Technology), PATRIC (The Pathosystems Resource Integration Center), and PGAP (Prokaryotic Genome Annotation Pipeline). Assembled genome sequence was submitted in RAST in FASTA format as input files, assigned functions to the genes. It also predicted the subsystems which were represented in the genome. By using this information, it reconstructs the metabolic network and makes the output file easily downloadable. Similarly, contigs were submitted in PATRIC as input files which provided annotation, subsystem summary, phylogenetic tree, and pathways. NCBI PGAP was used to annotate the bacterial genome where the complete genomic sequence was submitted in FASTA format as an input file, and it predicted the protein-coding regions and functional genome units like tRNAs, rRNA, pseudogenes, transposons, and mobile elements.

### Prediction of essential bacterial genes

The Geptop 2.0 web server provides an online platform tool to detect essential genes set across bacterial species using http://guolab.whu.edu.cn/geptop/. The nitrogen metabolism gene nucleotides (assimilatory nitrate reductase, respiratory nitrate reductase, nitrite reductase, nitric oxide reductase, hydroxylamine reductase, and glutamine synthetase) of bacteria *Lelliottia amnigena* were submitted in FASTA format. The web server computed the essentiality score of each gene by comparing Orthology and Phylogeny information (DEG database). The default essentiality cut-off was set at 0.24.

### Alignment of multiple gene sequences and phylogenetic analysis

N-metabolic genes were selected, and alignment of these genes was performed. The top three similar gene sequences of nitrate assimilation and denitrification were retrieved after doing BLASTP against the NCBI Nr database for the sequence alignment. Sequence Manipulation Suite version 2 was used for alignment and polished the protein sequences [20]. All the protein sequences of N-metabolism genes (assimilatory and respiratory nitrate reductase, nitrite reductase, nitric oxide reductase, hydroxylamine reductase, and glutamine synthetase) of *Lelliottia amnigena* and their similarities genes were analyzed by BLASTP and saved in FASTA format as an input file. To investigate the phylogenetic relationship of selected nitrogen metabolism genes was performed with the help of the MEGA 11(Mega Evolutionary Genetic Analysis version 11) tool. First, the protein sequence was aligned with MUSCLE and phylogenetic tree was constructed based on neighbor-joining [21]. The percentage of bootstrap [22] values were shown at the nodes. The evolutionary distances were computed using the Jones Taylor Thornton method [23] and are in the units of the number of amino acids substitutions per site. Branch length are given below the node. It defines the genetic changes i.e., longer the branch more genetic changes.

### 3D protein structure prediction

The putative 3D structure of the protein was predicted by I-TASSER software. It is a reliable tool for identifying the putative protein structure. This tool performs its work by threading to identify the template protein database that resembles the predicted protein and the fragments used to construct the protein model. The confident (C-value), TM score and RMSD from the i-TASSER result were used in predicting the protein model [24, 25].

### Metabolic pathway mapping in *Lelliottia amnigena* PTJIIT1005

To analyze the genes involved in the N-metabolism pathway predicted using PATRIC’s comparative pathway tool. All pathway comes from the Kyoto Encyclopedia of Genes and Genomes (KEGG) [26, 27]. It identifies metabolic pathways based on taxonomy, EC number, pathway ID, and Pathway name. A complete genome sequence was submitted in PATRIC to identify functional metabolic pathways in bacterial strains (Wattam et al. 2014).

## Results

### Genome sequencing and bioinformatic evaluation of *L. amnigena* PTJIIT1005

The genome of *L. amnigena* PTJIIT1005 was sequenced on the NovaSeq6000 Illumina platform by Redcliffe Lifetech Pvt. Ltd., Noida. A total of 8,596,940 high-quality reads were generated. The estimated bacterial genome size is ~ 4.5 Mb. The characteristics and properties of genome sequence assembly are shown in Table 1.

**Table 1:** *Lelliottia amnigena* PTJIIT1005 genome assembly summary

Total no. of scaffold obtained	85
Total size of the scaffolds (in megabases)	4,552,797(~ 4.5 Mb)
Min. scaffold length (bp)	100
Max. scaffold length (bp)	1,125,792
Average scaffold length (bp)	53,562.3
Protein coding genes	4259
GC content %	52.92
tRNA	76

The raw data and assembled genome sequence were submitted to NCBI GenBank (Accession no. PRJNA830830 and JAKRZN000000000 respectively), PATRIC (ID 61646.105), and RAST (ID 806758.3).

**Fig. 1:**
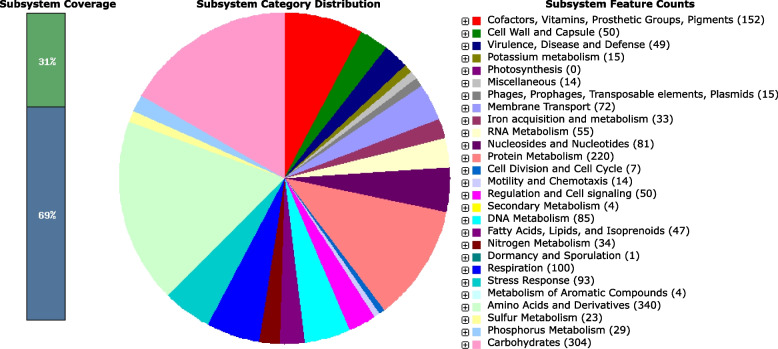
Distribution of genes present in subsystem by RAST. The upper value of the bar graph represents the percentage of present genes, and the lower value represents the percentage of absent genes in the subsystem

The Nucleotide Identity (ANI) [17] analysis of PTJIIT1005 with closely related species exhibited a similarity index (98.89%) with the reference genome *Lelliottia amnigena* NZ_CP077236.1. Total of 4365 genes was annotated in the genome of *L. amnigena* PTJIIT1005 by NCBI Prokaryotic Genome Annotation Pipeline (PGAP), including 4275 CDS, one ribosomal RNA, 73 tRNA, and 68 pseudogenes. PATRIC gives a total of 4423 CDS, 74 tRNA with 71contigs. SEED subsystem based RAST annotation gives a total of 4424 coding genes involving 77 RNAs. RAST server SEED VIEWER clearly showed the subsystem coverage, division, and the number of coding regions present in subsystem. Coding sequencing comprise of 359 subsystems (sets of functionally related protein families). A total of 4424 coding regions, out of these, 1353 were present in subsystem; non-hypothetical 1306, hypothetical-47 and left 3071 CDS not present in any subsystem; non-hypothetical 2039, hypothetical 1032 as shown in Fig. 1. Each subsystem which are present in pie chart is shown in Supplementary material (as Supplementary Table 1) with all details like number of genes and percentage values.

### Identification of N-metabolism gene sequence

Genome and functional annotation define the structural and functional information of genes that helped to select N-metabolism genes involved in nitrification, denitrification, and N-assimilation. The PATRIC tool, RAST, and PGAP (Prokaryotic Genome Annotation Pipeline) were used to annotate the N-metabolic genes revealed Hydroxylamine reductase (550 residues), nitrite reductase (955 residues), assimilatory nitrate reductase (839 residues), respiratory nitrate reductase including alpha, beta, gamma and delta (2217residues), nitric oxide reductase (377 residues) and glutamine synthetase I (469 residues).

**Fig. 2:**
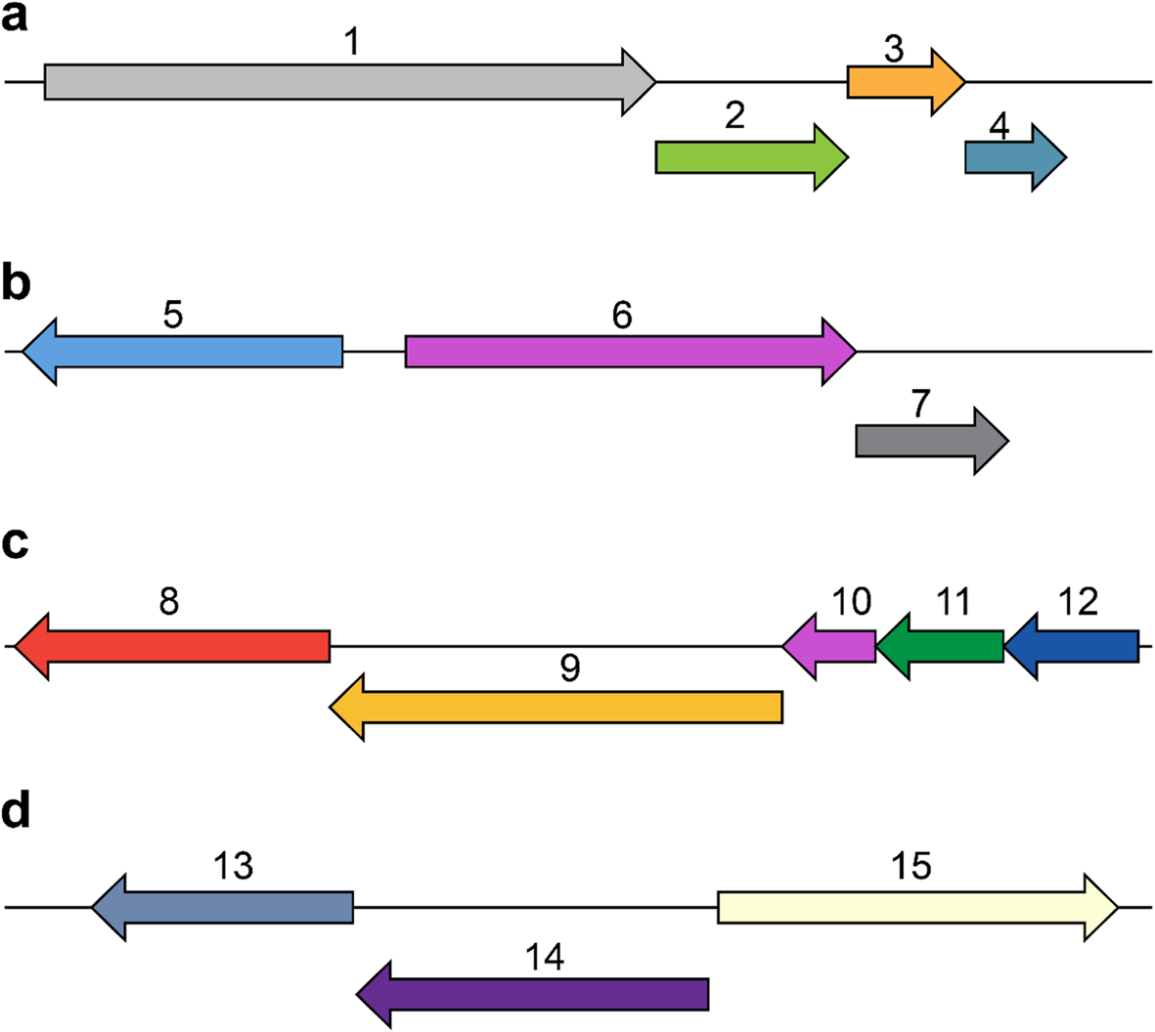
Representation of bacterial genes *L. amnigena* PTJIIT1005 genome predicted by PATRIC [28] and RAST server. Arrow size is related to gene size. **a**. These N-metabolic genes are located on contig 6. Arrow 1–4 NarGHJI illustrates operon for respiratory nitrate reductase subunit, alpha, beta, gamma, and delta. **b**. Arrow 5, representing nitrate/ nitrite transporter, Nark, Arrow 6, nitrate/nitrite sensor protein, NarX, Arrow 7, nitrate/nitrite response regulator protein, NarL. **c**. Arrows 8 and 9 indicate assimilatory nitrate and nitrite reductase, large subunit, respectively, and arrow 10–12, operon for nitrate ABC transporter, involving genes for ATP-binding protein, permease protein, and substrate binding, respectively. **d**. Nitric oxide reductase gene located on contig 8, arrow 13, nitric oxide reductase FlRd-NAD reductase, arrow 14, anaerobic nitric oxide reductase transcription regulator, arrow 15, anaerobic nitric oxide reductase flavorubredoxin

PATRIC tool annotates different genes related to nitrogen metabolism. Figure 2(a) shows the operon NarGHJI for respiratory nitrate reductase on contig 6.

To the upstream of an operon, NarGHJI represented the gene for nitrate/ nitrite transporter, sensor, and regulator protein (Fig. 2b). Operon for assimilatory nitrate and nitrite reductase, nitrate ABC transporter involves genes for ATP-binding protein, permease protein, and substrate binding on the same contig (Fig. 2c). Similarly, Fig. 2d shows that genes related to nitrosative stress include nitic oxide reductase Fl-Rd-NAD (+) reductase, anaerobic nitric oxide reductase flavorubredoxin, anaerobic nitric oxide reductase transcription regulator NorR on contig 8.

### Predicted N-metabolism essential genes

We predicted 397 essential genes out of total of 4340 genes using the Geptop method. Among all N-metabolism genes, only glutamine synthetase is an essential gene and has an essentiality score of 0.3881.

### Multiple gene sequence alignment and phylogenetic trees

**Fig. 3:**
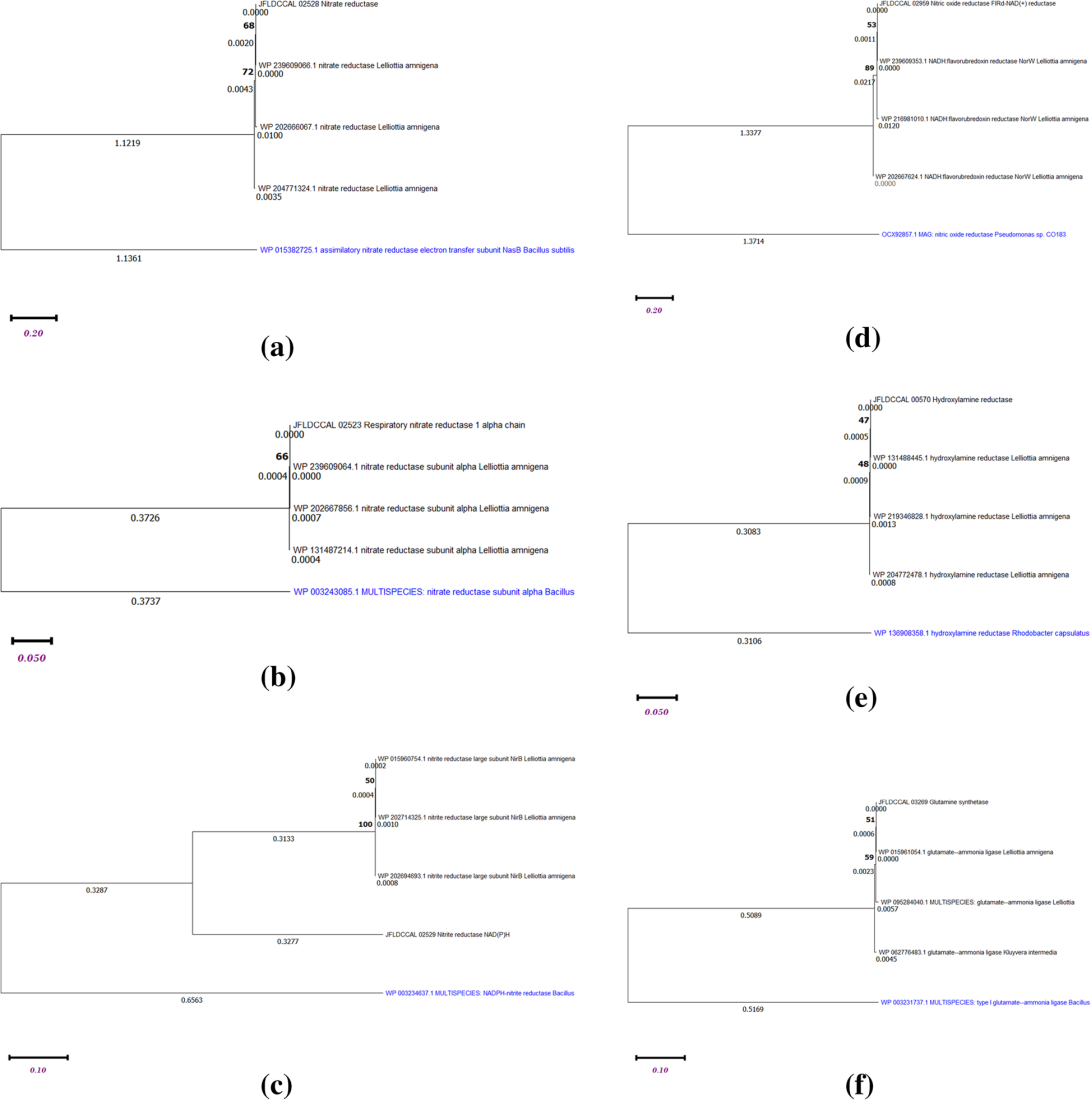
Phylogenetic tree was constructed based on neighbor-joining using MEGA 11.0.11 version software with 100 bootstrap replications. The value of bootstrap replicates is shown at the nodes and blue marked as an outgroup. **a**. Phylogenetic tree of assimilatory nitrate reductase; JFLDCCAL 02528- an isolated *L. amnigena* strain similar to the top three NCBI strains whose accession no. WP239609066, WP204771324, WP202666067; *Bacillus subtilis* (WP015382725) is used as out group reference (blue marked). Bootstrap value is indicated on nodes (in bold), branch length value below the node, and scale bar value indicates 0.20 substitutions per nucleotide position. **b**. Phylogenetic tree of respiratory nitrate reductase; JFLDCCAL02523 nitrate reductase- an isolated *L. amnigena* strain is similar to top three NCBI strain whose accession numbers are WP239609064, WP202667856, WP 131487214; Bacillus strain (WP003243085) is used as an out group reference (blue marked). Bootstrap value is indicated on nodes (in bold), branch length value below the node and scale bar value indicates 0.050 substitutions per nucleotide position. **c**. Phylogenetic tree of nitrite reductase; JFLDCCAL02529 nitrite reductase- an isolated *L. amnigena* strain is similar to top three accession numbers WP015960754, WP202714325 and WP202694693; Bacillus strain (WP003234637) is used as an outgroup reference (blue marked). Bootstrap value is indicated on nodes (in bold), branch length value below the node and scale bar value indicates 0.10 substitutions per nucleotide position. **d**. Phylogenetic tree of nitric oxide reductase; JFLDCCAL02959-an isolated *L. amnigena* strain similar to top three strains whose accession no. WP239609353, WP202667624, WP216981010; Pseudomonas sp. (OCX92857) is used as an outgroup reference (blue marked). Bootstrap value is indicated on nodes (in bold), branch length value below the node and scale bar value indicates 0.20 substitutions per nucleotide position. **e**. Phylogenetic tree of hydroxylamine reductase; JFLDCCAL 00570- an isolated *L. amnigena* strain is similar to top three strains accession no. WP204772478, WP219346828 and WP131488445; *Rhodobacter capsulatus* strain is used as an outgroup reference (blue marked). Bootstrap value is indicated on nodes (in bold), branch length value below the node and scale bar value indicates 0.050 substitutions per nucleotide position. **f**. Phylogenetic tree of glutamine synthetase, JFLDCCAL03269- an isolated *L. amnigena* strain similar to top three strains WP015961054, WP062776483, WP095284040; Bacillus strain is used as an outgroup reference (blue marked). Bootstrap value is indicated on nodes (in bold), branch length value below the node and scale bar value indicates 0.10 substitutions per nucleotide position

BLASTP was used to align multiple gene sequences of interesting genes with three closely related genes. The detailed result of the protein sequence alignment of N-metabolic genes are shown in Table 2. For the result of multiple alignments of nitrogen genes, please refer to the supplementary material (S1-S10). A phylogenetic analysis of gene sequences exhibits a percentage of higher resemblance with strain *L. amnigena*, as shown in Fig. 3. The trees were constructed using MEGA11 software with 100 bootstrap replications, the percentage values of bootstrap are indicated at the nodes. The analysis involved 5 amino acids sequences. Branch length are given below the node. Scale bar refers to a phylogenetic distance of value (0.20, 0.10 and 0.050) nucleotide substitutions per site. The alignment report and phylogenetic trees of these nitrogen metabolic genes evidently showing strain specific resemblance and distinction.

**Table 2:** Alignment of protein sequences of *Lelliottia amnigena* PTJIIT1005

N-metabolic pathway genes	Top most (3) BLAST hits	E-value	Similarity percentage (%)
Assimilatory nitrate reductase (nasA)	*L*. *amnigena* WP_239609066,	0.0	839/839 (100%)
	*L. amnigena* WP_204771324	0.0	830/839 (98.93%)
	*L. amnigena* WP_202666067	0.0	829/839 (98.81%)
Respiratory nitrate reductase (narG)	*L. amnigena* WP_239609064	0.0	1246/1246 (100%)
	*L. amnigena* WP_131,487,214	0.0	1245/1246 (99.92%)
	*L. amnigena* WP_202667856	0.0	1245/1246 (99.91%)
Nitrite reductase (NirB)	*L. amnigena* WP_015960754	0.0	847/847 (100%)
	*L. amnigena* WP_202694693	0.0	845/847 (99.88%)
	*L. amnigena* WP_202714325	0.0	845/847 (99.88%)
Nitric oxide reductase (NorW)	*L. amnigena* WP_239609353	0.0	377/377 (100%)
	*L. amnigena* WP_202667624	0.0	371/377 (98.41%)
	*L. amnigena* WP_216981010	0.0	371/377 (98.67%)
Hydroxylamine reductase	*L. amnigena WP_131488445*	0.0	550/550 (100%)
	*L. amnigena WP_204772478*	0.0	549/550 (99.82%)
	*L. amnigena WP_219346828*	0.0	549/550 (99.82%)
Glutamine synthetase I (GlnA)	*L. amnigena* WP_015961054	0.0	469/469 (100%)
	*Lelliottia* WP_095284040	0.0	465/469 (99.36%)
	*Kluyvera intermedia* WP_062776483	0.0	465/469 (99.15%)

### Site of N-metabolism-related genes in KEGG pathway

**Fig. 4:**
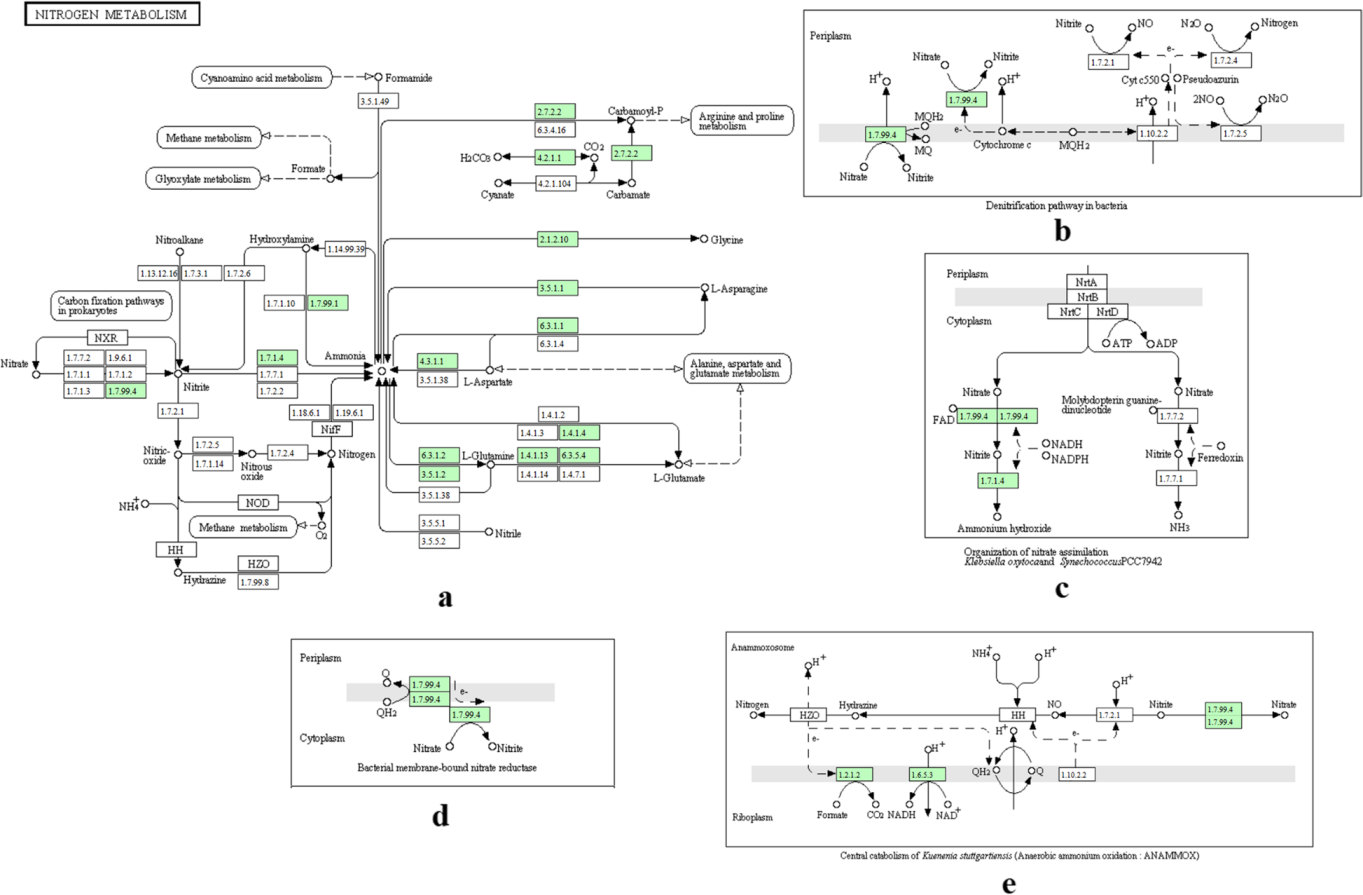
**a**: Prediction of nitrogen metabolic pathway for *Lelliottia amnigena* PTJIIT1005 using PATRIC KEGG feature [26] [29]. The green shaded box represents the respective gene in a given metabolic pathway, and it consists of three nitrogen reactions like denitrification, N-assimilation, and anaerobic ammonia oxidation (nitrification) **b**: Represent the denitrification step in bacteria, **c**: Represent the N-assimilation reaction, **d**: Represent the membrane-bound nitrate reductase enzyme, **e**: Represent the anaerobic ammonia oxidati*on, also known as nitrification*

The KEGG pathway described the metabolic features of *Lelliottia amnigena* PTJIIT1005 (Fig. 4). Entire N-metabolism was analyzed to assess the potential of bacteria like N-assimilation, anaerobic ammonia oxidation (nitrification), and denitrification reactions. PATRIC could not map nitric oxide reductase enzyme in the pathway. As seen in Fig. 4, PATRIC mapped nitrate reductase (E.C. 1.7.99.4), nitrite reductase (E.C. 1.7.1.4), hydroxylamine reductase (E.C.1.7.99.1) and glutamine synthetase (E.C. 6.3.1.2) genes, indicated in green color. In the nitrogen assimilation pathway, nitrate is transformed into nitrite by nitrate reductase and nitrite into ammonia by nitrite reductase. In the end, ammonia is converted into glutamine by glutamine synthetase, which bacteria assimilate it into cell biomass for growth. Nitrate and nitrite can act as an alternative to oxygen under anoxic conditions. Compared to the N-assimilation pathway, nitrogen as nitrate is lost in the form of nitrogen, i.e., denitrification. In denitrification, the respiratory nitrate reductase gene is involved. Additionally, the PATRIC server annotated the presence of nitric oxide reductase on contigs 8 relating to the nitrosative stress subsystem. It represents the capability of the bacterium for nitric oxide reducing function.

### Prediction of 3D protein structures

**Fig. 5:**
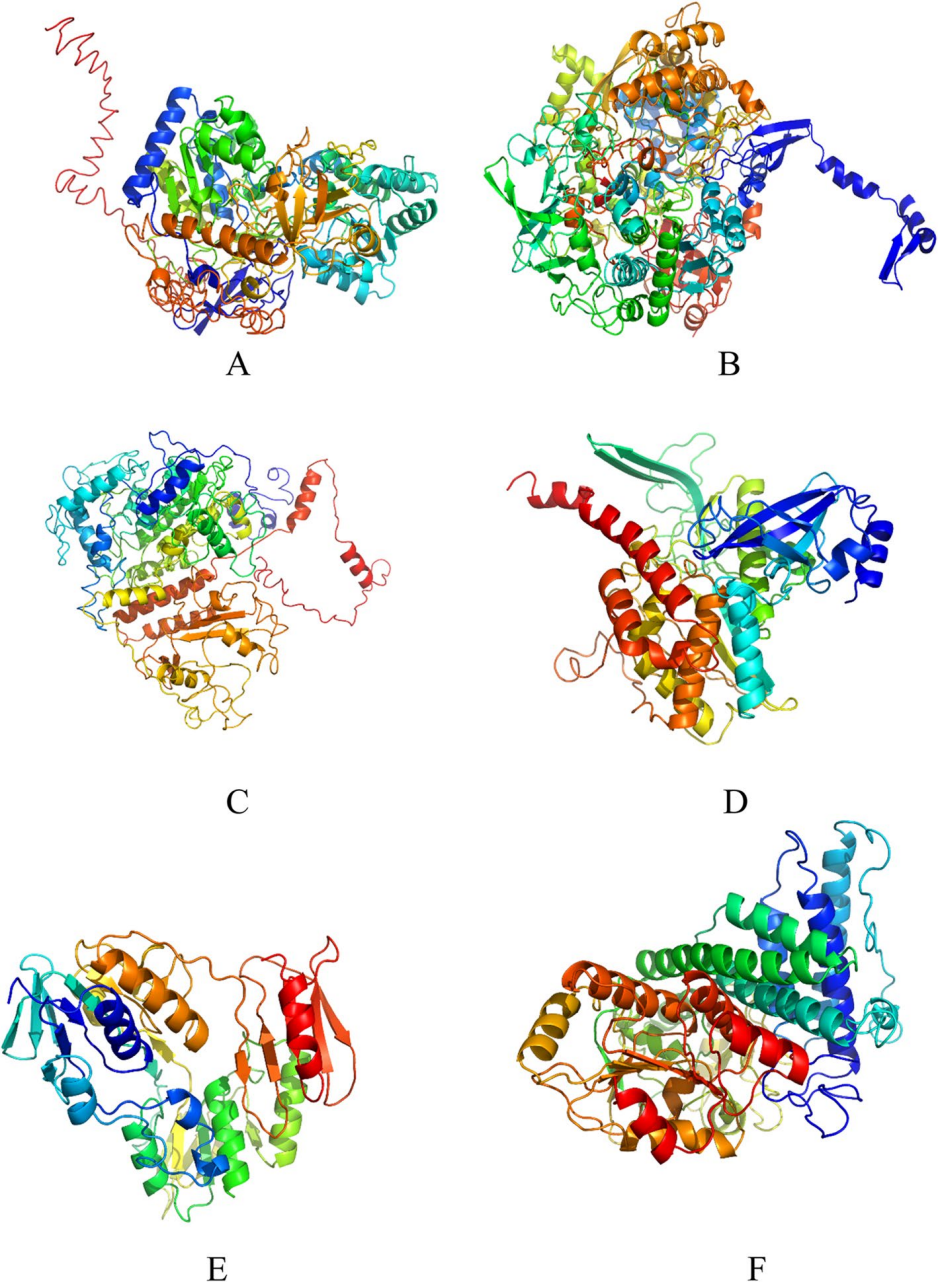
3D protein modelling of (**A**) Assimilatory nitrate reductase predicted by I-TASSER (**B**) Respiratory nitrate reductase (**C**) Nitrite reductase (**D**) Glutamine synthetase (**E**) Nitric oxide reductase (**F**) Hydroxylamine reductase

I-TASSER was used for 3D protein structure prediction, generating C-score, TM score, and RMSD values. C-score is a confidence score for determining predicted models quality. The higher the C-score value, the higher confidence of protein structure. TM scores and RMSD values were used to analyze the structure similarity between the reference templates and predicted proteins. The highest TM score means a high structural similarity. Predicted protein models of all N-metabolisms genes are shown in Fig. 5.

The statistical results of all predicted protein structures are shown in Table 3. As per the table, N-metabolism genes such as respiratory nitrate reductase, glutamine synthetase, hydroxylamine reductase, and nitric oxide reductase have positive C-value means good quality of predicted models and its TM score & RMSD value showing higher similarity with the templates except for assimilatory nitrate reductase and nitrite reductase (negative C-value and less similarity with template).

**Table 3:** Statistical outcomes of all predicted protein models

N-metabolism genes	C-value	TM score	Root Mean Square Deviation (RMSD) in Å
Assimilatory nitrate reductase	−2.20	0.45	14.0
Respiratory nitrate reductase	1.97	0.99	5.1
Nitrite reductase	−1.47	0.53	12.1
Glutamine synthetase	1.50	0.92	4.0
Hydroxylamine reductase	1.71	0.95	3.9
Nitric oxide reductase	0.72	0.81	5.2

## Discussion

Genes involved in N-metabolic pathways are assimilatory nitrate reductase (nas), respiratory nitrate reductase (nar), nitrite reductase (nir), nitric oxide reductase (nor), hydroxylamine reductase (har), and glutamine synthetase (gln). These are N-assimilation and denitrification pathways which responsible for nitrogen removal from media.

In N-assimilation, nitrate is first transformed into nitrite and ammonia and further converted into glutamine. Glutamine assimilates by microbes to increase the biomass [30, 31]. In denitrification, nitrate is converted into nitrite, nitrite into nitric oxide, nitric oxide into nitrous oxide, and finally into nitrogen [32, 33].

*L. amnigena* PTJIIT1005 shows promising growth in the presence of nitrate and simultaneously remediated nitrate [34]. A coupled biochemical reaction of nitrification and denitrification is involved in the nitrogen removal process. So, we emphasize whole-genome sequencing of this bacteria to identify N-assimilation, nitrification, and denitrification-related genes. Genome sequencing was analyzed by NGS (Next Generation Sequencing), and the genome was annotated by the PGAP pipeline, PATRIC, and RAST server. N-assimilation, nitrification, and denitrification genes were targeted to see their importance in removing nitrate from the media.

In N-assimilation, the nitrate ABC transporter is involved in the nitrate influx into the cells [35, 36]. Annotation of all these three genes (Fig. 2c) related to periplasmic nitrate binding protein, ATP binding protein, and permease protein, respectively. These genes demonstrate the presence of a nitrate assimilation system in *Lelliottia amnigena* PTJIIT1005. Afterward, the nitrate reductase, nitrite reductase, and glutamine synthetase genes were annotated and exhibited the entire N-assimilatory pathway. In contrast, denitrification is incomplete because of lacking nitrous oxide reductase in this genome assembly.

Different bioinformatics tools were used to pick up the desired N-genes. RAST and PATRIC tools were used to identify the N-metabolic genes and the genes’ location. N-assimilatory and denitrification genes were found on the same contig 6 (Fig. 2a and c). The nitrosative stress subsystem on contig 8 suggests that it only operates in stress conditions like oxygen-limiting. It gives facultative bacteria the capability to utilize nitrate as an electron acceptor.

There is also a nitrification step in this bacteria, i.e., ammonia oxidation, which also occurs under anoxic conditions. But this process is incomplete due to the absence of ammonia monooxygenase enzyme [37, 38].

Multiple alignments of protein sequence outcomes showed that assimilatory and respiratory nitrate reductase, nitrite reductase, nitric oxide reductase, glutamine synthetase, and hydroxylamine reductase are highly conserved among microbial strains belonging to *Lelliottia amnigena*. They exhibited 98–100% sequence similarity with *L. amnigena* strains. Further, these proteins were examined to analyze the 3D protein models. I-TASSER was used to generate a 3D protein model; according to this tool, all genes have found good quality protein models and good sequence identity with reference templates available in PDB except assimilatory nitrate reductase and nitrite reductase.

Genes were mapped on the nitrogen metabolic pathway using the PATRIC KEGG feature when selected. They were found and belong to N-assimilation, denitrification, and anammox chemical reactions. Moreover, this bacteria might have an alternative N-removal pathway where hydroxylamine is an intermediate for further denitrification reactions. This type of heterotrophic denitrification has been found in strain *Acinetobacter calcoaceticus* HNR [39, 40].

All these features make this bacteria a promising candidate in assisting inorganic N-removal from contaminated water.

## Conclusion

In our paper, the bacterial strain *L. amnigena* PTJIIT1005 isolated from the polluted site of Yamuna River was efficient in nitrate remediation. Complete genome sequence analysis confirms the presence of N-metabolism genes in the bacterial strain. All this information could help further optimize the production of bacterial enzymes and their usage in wastewater treatment.

## Supplementary Information

**Additional file 1: Table S1.** This table shows connection between pie chart and subsystem lists by indicating each subsystems, number of genes and their percentage value. **Fig. S1*.*** Multiple sequence alignment of assimilatory nitrate reductase gene from *Lelliottia amnigena﻿* PTJIIT1005 (JAKRZN000000000) with similar strains *L. amnigena* WP_239609066, *L. amnigena* WP_204771324, and *L. amnigena* WP_202666067.Consensus sequence marked with black color and non-consensus marked with white. **Fig. S2*.*** Multiple gene sequence alignment of respiratory nitrate reductase alpha subunit of *L. amnigena* PTJIIT1005 (JAKRZN000000000) similar to strains *L. amnigena* WP_239609064, *L. amnigena* WP_131,487,214, and *L. amnigena* WP_202667856. Consensus sequence marked with black color and non-consensus marked with white. **Fig. S3*.*** Multiple sequence alignment of respiratory nitrate reductase beta subunit of *Lelliottia amnigena* PTJIIT1005 (JAKRZN000000000) show similarity with *L. amnigena* WP_059179798, *L. amnigena* WP_202666064, and *L. amnigena* WP_216981296. Consensus sequence marked with black color and non-consensus marked with white. **Fig. S4*.*** Multiple sequence alignment of respiratory nitrate reductase gamma subunit of *Lelliottia amnigena* PTJIIT1005 (JAKRZN000000000) similar to Enterobacteriaceae WP_123754749, *L. amnigena* WP_059179800 and *L. amnigena* WP_131487216. Consensus sequence marked with black color and non-consensus marked with white. **Fig. S5*.*** Multiple sequence alignment of respiratory nitrate reductase delta subunit of *Lelliottia amnigena* PTJIIT1005 (JAKRZN000000000) similar to *L. amnigena* WP_235135660, Enterobacteriaceae WP_047051075 and Enterobacteriaceae WP_181623582. Consensus sequence marked with black color and non-consensus marked with white. **Fig. S6*.*** Multiple sequence alignment of nitrite reductase large subunit [NAD(P)H] of *Lelliottia amnigena* PTJIIT1005 (JAKRZN000000000) similar to *L*. *amnigena* WP_015960754, *L. amnigena* WP_202694693, and *L. amnigena* WP_202714325. Consensus sequence marked with black color and non-consensus marked with white. **Fig. S7*.*** Multiple sequence alignment of nitrite reductase small subunit [NAD(P)H] of *Lelliottia amnigena* PTJIIT1005 (JAKRZN000000000) similar to *L. amnigena* WP_015960755, *L. amnigena* WP_252686794, and Enterobacter sp. MCI1899116. Consensus sequence marked with black color and non-consensus marked with white. **Fig. S8*.*** Multiple sequence alignment of glutamine synthetase I of *Lelliottia amnigena* PTJIIT1005 (JAKRZN000000000) similar to *Lelliottia amnigena* WP_015961054, *Lelliottia* WP_095284040, and *Kluyvera intermedia* WP_062776483. Consensus sequence marked with black color and non-consensus marked with white. **Fig. S9*.*** Multiple sequence alignment of nitric oxide reductase of *Lelliottia amnigena* PTJIIT1005 (JAKRZN000000000) similar to *Lelliottia amnigena* WP_239609353, *Lelliottia* WP_202667624, and *L. amnigena* WP_216981010. Consensus sequence marked with black color and non-consensus marked with white. **Fig. S10.** Multiple sequence alignment of hydroxylamine reductase of *Lelliottia amnigena* PTJIIT1005 (JAKRZN000000000) similar to *Lelliottia amnigena* WP_131488445, *Lelliottia* WP_204772478, and *Lelliottia* WP_219346828. Consensus sequence marked with black color and non-consensus marked with white.

## Data Availability

The complete genome sequence has been deposited to NCBI GenBank under the accession number JAKRZN000000000 (BioProject: PRJNA806758, BioSample: SAMN25898493). The raw reads also have been deposited in Sequence Read Archive (SRA) under BioProject accession number PRJNA830830.
