# Supplementary material for: Identification and examination of nitrogen metabolic genes in Lelliottia amnigena PTJIIT1005 for their ability to perform nitrate remediation
Source: BMC Genomics. 2023 Mar 9;24:104. doi: 10.1186/s12864-023-09207-6 (PMC9999607; doi:10.1186/s12864-023-09207-6)
Supplement: Supplementary file 1 — Additional file 1: Table S1. This table shows connection between pie chart and subsystem lists by indicating each subsystems, number of genes and their percentage value. Fig. S1. Multiple sequence alignment of assimilatory nitrate reductase gene from Lelliottia amnigena﻿ PTJIIT1005 (JAKRZN000000000) with similar strains L. amnigena WP_239609066, L. amnigena WP_204771324, and L. amnigena WP_202666067.Consensus sequence marked with black color and non-consensus marked with white. Fig. S2. Multiple gene sequence alignment of respiratory nitrate reductase alpha subunit of L. amnigena PTJIIT1005 (JAKRZN000000000) similar to strains L. amnigena WP_239609064, L. amnigena WP_131,487,214, and L. amnigena WP_202667856. Consensus sequence marked with black color and non-consensus marked with white. Fig. S3. Multiple sequence alignment of respiratory nitrate reductase beta subunit of Lelliottia amnigena PTJIIT1005 (JAKRZN000000000) show similarity with L. amnigena WP_059179798, L. amnigena WP_202666064, and L. amnigena WP_216981296. Consensus sequence marked with black color and non-consensus marked with white. Fig. S4. Multiple sequence alignment of respiratory nitrate reductase gamma subunit of Lelliottia amnigena PTJIIT1005 (JAKRZN000000000) similar to Enterobacteriaceae WP_123754749, L. amnigena WP_059179800 and L. amnigena WP_131487216. Consensus sequence marked with black color and non-consensus marked with white. Fig. S5. Multiple sequence alignment of respiratory nitrate reductase delta subunit of Lelliottia amnigena PTJIIT1005 (JAKRZN000000000) similar to L. amnigena WP_235135660, Enterobacteriaceae WP_047051075 and Enterobacteriaceae WP_181623582. Consensus sequence marked with black color and non-consensus marked with white. Fig. S6. Multiple sequence alignment of nitrite reductase large subunit [NAD(P)H] of Lelliottia amnigena PTJIIT1005 (JAKRZN000000000) similar to L. amnigena WP_015960754, L. amnigena WP_202694693, and L. amnigena WP_202714325. Consensus sequence [file 12864_2023_9207_MOESM1_ESM.docx]

| **Subsystems** | **Number of genes** | **Percentage (%)** |
| --- | --- | --- |
| Cofactors, Prosthetic groups, Pigments | 152/1891 | 8.03 |
| Cell wall and capsule | 50/1891 | 2.644 |
| Virulence, Disease & Defense | 49/1891 | 2.59 |
| Potassium metabolism | 15/1891 | 0.79 |
| Miscellaneous | 14/1891 | 0.74 |
| Phages, Prophages, Transposable elements, Plasmids | 15/1891 | 0.793 |
| Membrane transport | 72/1891 | 3.80 |
| Iron acquisition & metabolism | 33/1891 | 1.745 |
| RNA metabolism | 55/1891 | 2.90 |
| Nucleosides and Nucleotides | 81/1891 | 4.28 |
| Protein metabolism | 220/1891 | 11.63 |
| Cell division & Cell cycle | 7/1891 | 0.370 |
| Motility and chemotaxis | 14/1891 | 0.740 |
| Regulation & cell signaling | 50/1891 | 2.644 |
| DNA Metabolism | 85/1891 | 4.49 |
| Fatty acids, lipids & Isoprenoids | 47/1891 | 2.485 |
| Nitrogen Metabolism | 34/1891 | 1.797 |
| Respiration | 100/1891 | 5.288 |
| Stress Response | 93/1891 | 4.91 |
| Amino acids and Derivatives | 340/1891 | 17.97 |
| Sulfur Metabolism | 23/1891 | 1.21 |
| Phosphorus Metabolism | 29/1891 | 1.53 |
| Carbohydrates | 304/1891 | 16.07 |
| Secondary Metabolism | 4/1891 | 0.211 |
| Dormancy and Sporulation | 1/1891 | 0.0528 |
| Metabolism of Aromatic compounds | 4/1891 | 0.211 |

Table S1: This table shows connection between pie chart and subsystem lists by indicating each subsystems, number of genes and their percentage value.

Multiple sequence alignment of genes


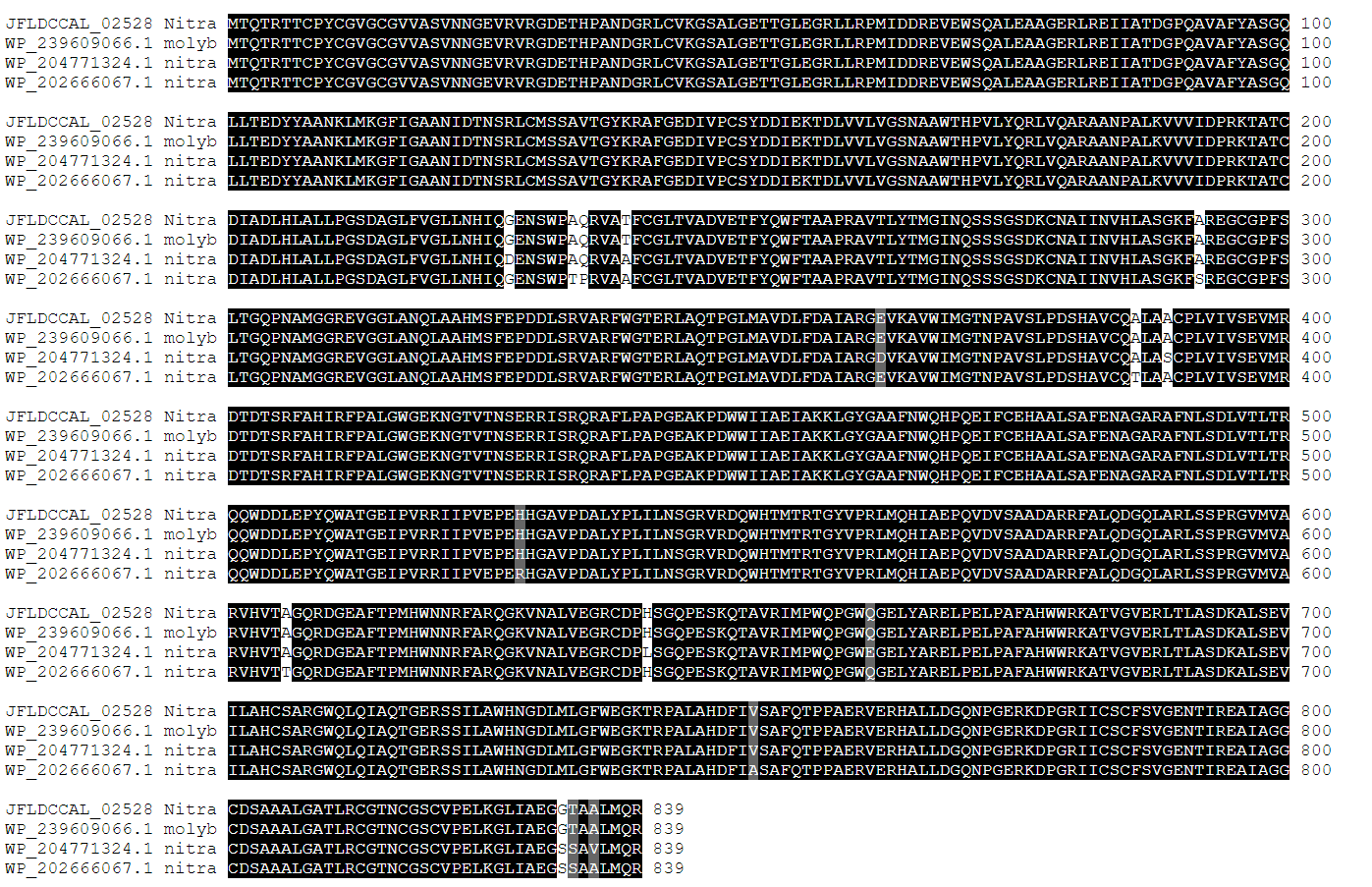


Figure S1: Multiple sequence alignment of assimilatory nitrate reductase gene from Lelliottia amnigena PTJIIT1005 (JAKRZN000000000) with similar strains L. amnigena WP_239609066, L. amnigena WP_204771324, and L. amnigena WP_202666067.Consensus sequence marked with black color and non-consensus marked with white.


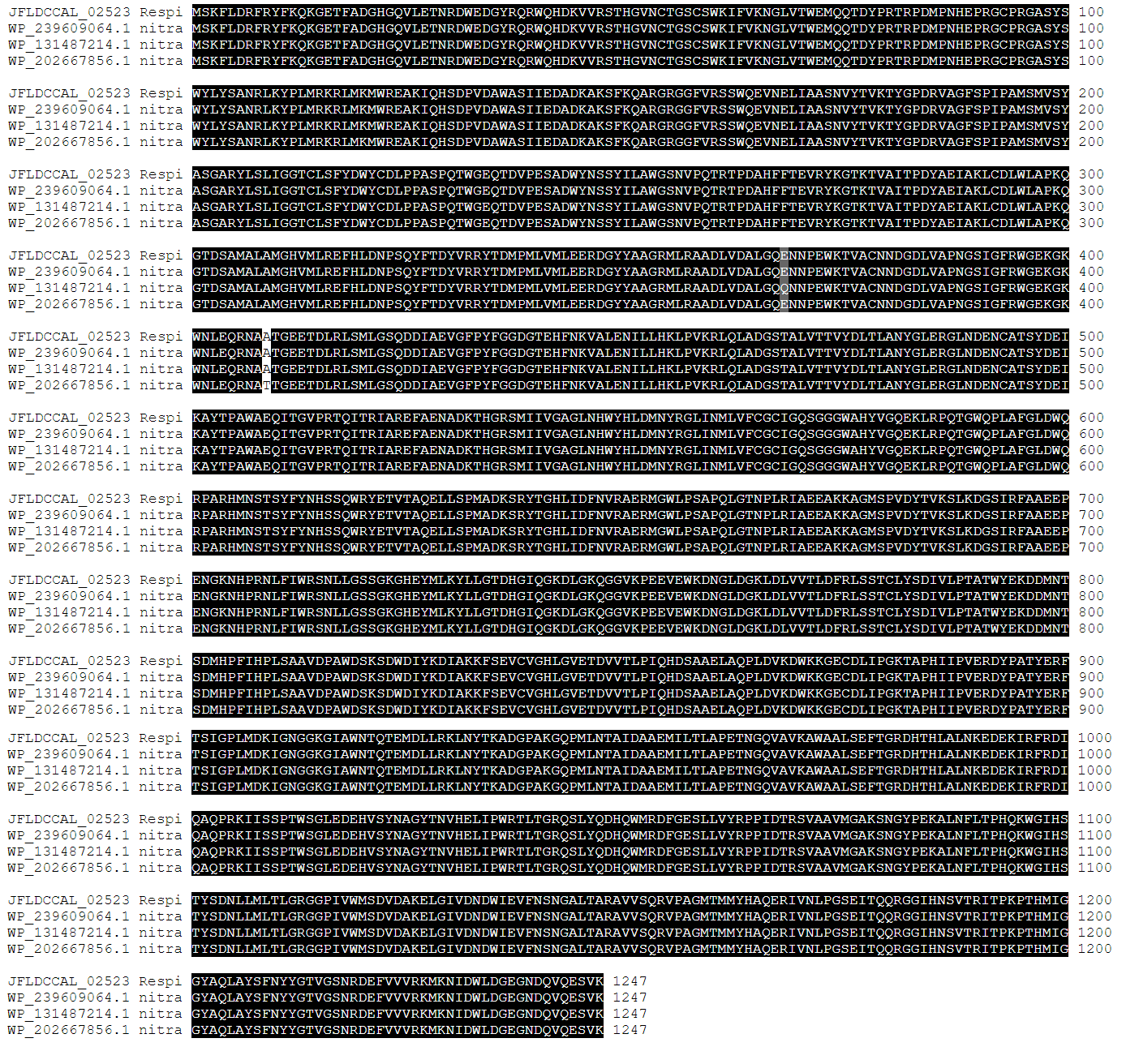


Figure S2: Multiple gene sequence alignment of respiratory nitrate reductase alpha subunit of L. amnigena PTJIIT1005 (JAKRZN000000000) similar to strains L. amnigena WP_239609064, L. amnigena WP_131487214, and L. amnigena WP_202667856. Consensus sequence marked with black color and non-consensus marked with white.


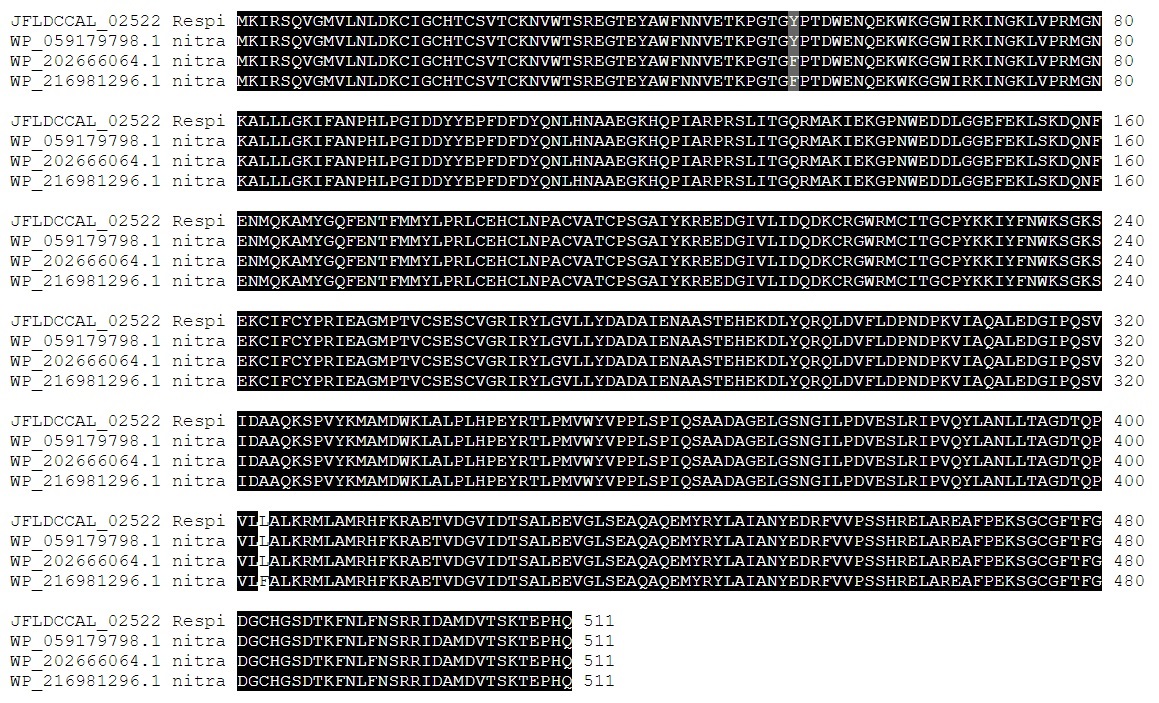


Figure S3: Multiple sequence alignment of respiratory nitrate reductase beta subunit of Lelliottia amnigena PTJIIT1005 (JAKRZN000000000) show similarity with L. amnigena WP_059179798, L. amnigena WP_202666064, and L. amnigena WP_216981296. Consensus sequence marked with black color and non-consensus marked with white.


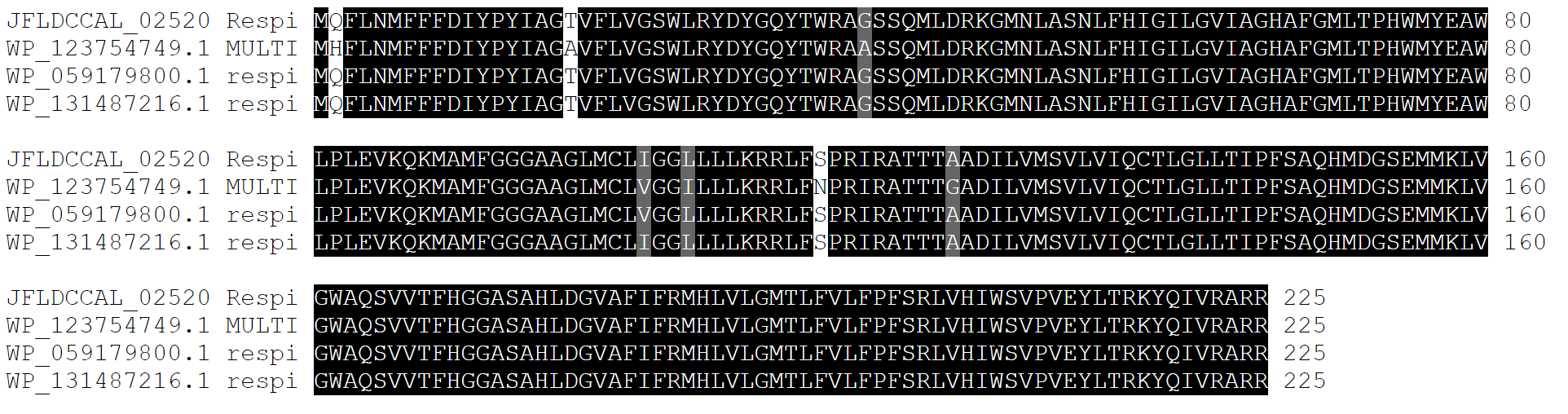


Figure S4: Multiple sequence alignment of respiratory nitrate reductase gamma subunit of Lelliottia amnigena PTJIIT1005 (JAKRZN000000000) similar to Enterobacteriaceae WP_123754749, L. amnigena WP_059179800 and L. amnigena WP_131487216. Consensus sequence marked with black color and non-consensus marked with white.


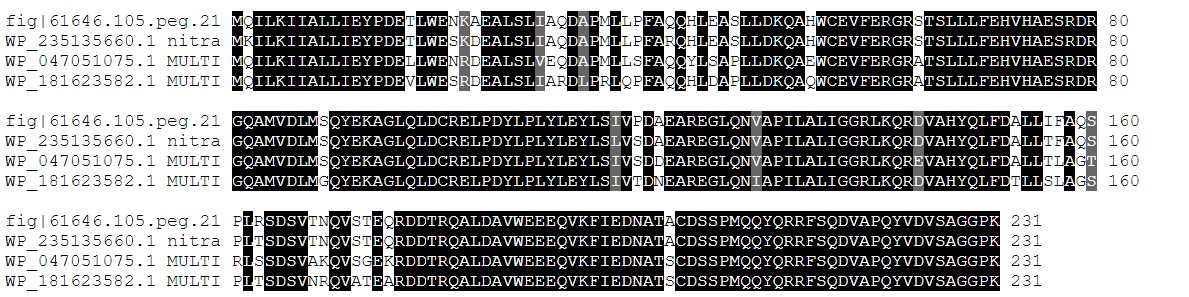


Figure S5: Multiple sequence alignment of respiratory nitrate reductase delta subunit of Lelliottia amnigena PTJIIT1005 (JAKRZN000000000) similar to L. amnigena WP_235135660, Enterobacteriaceae WP_047051075 and Enterobacteriaceae WP_181623582. Consensus sequence marked with black color and non-consensus marked with white.


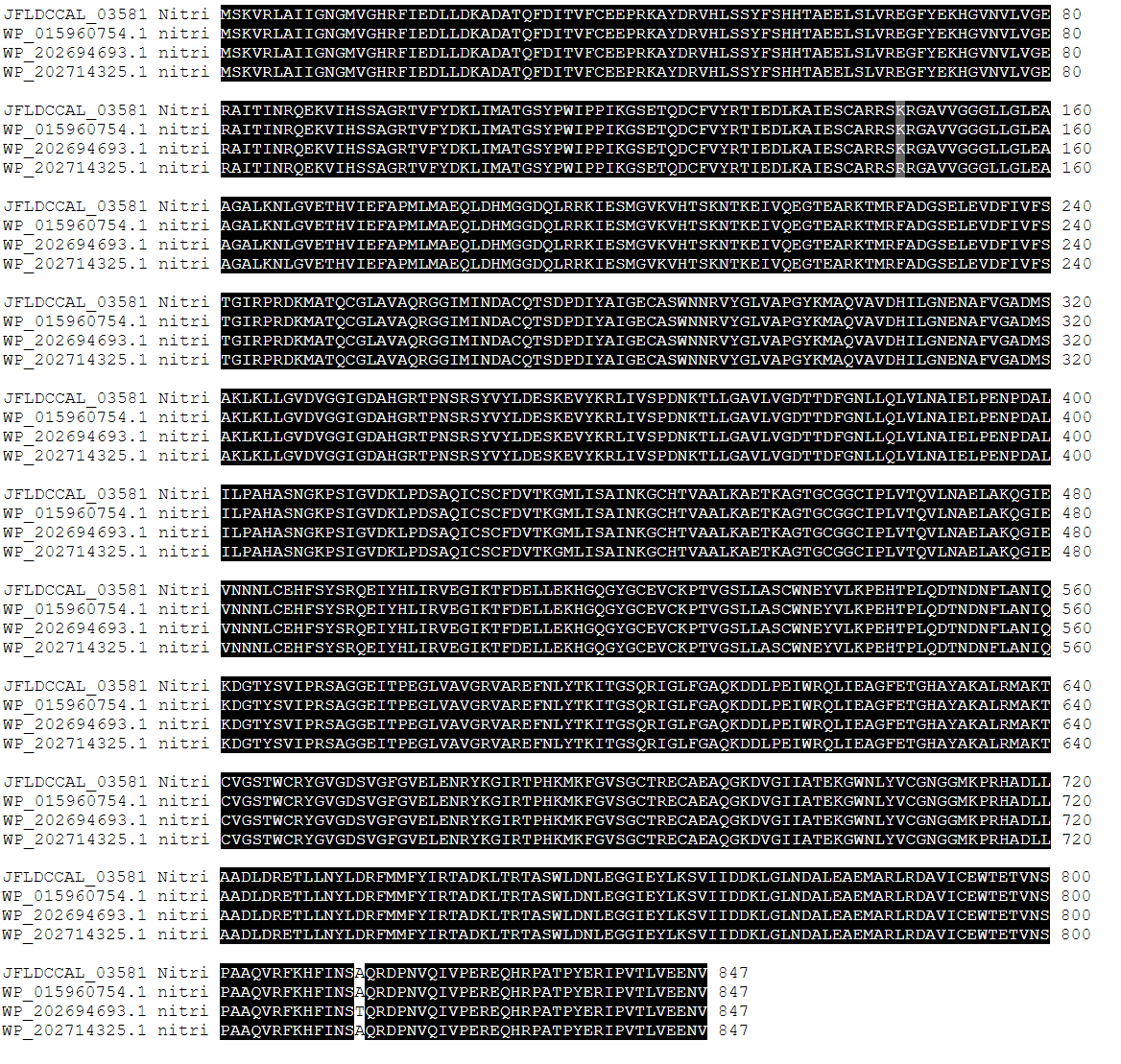


Figure S6: Multiple sequence alignment of nitrite reductase large subunit [NAD(P)H] of Lelliottia amnigena PTJIIT1005 (JAKRZN000000000) similar to L. amnigena WP_015960754, L. amnigena WP_202694693, and L. amnigena WP_202714325. Consensus sequence marked with black color and non-consensus marked with white.


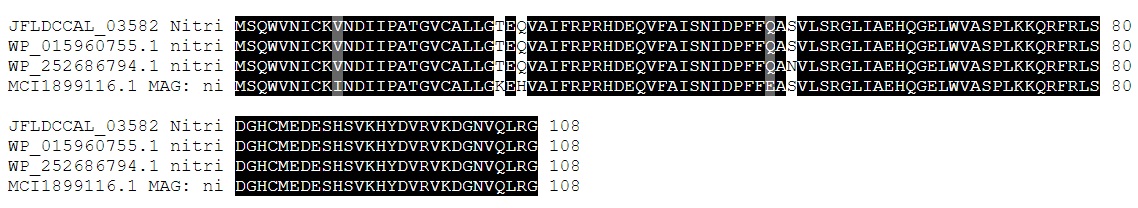


Figure S7: Multiple sequence alignment of nitrite reductase small subunit [NAD(P)H] of Lelliottia amnigena PTJIIT1005 (JAKRZN000000000) similar to L. amnigena WP_015960755, L. amnigena WP_252686794, and Enterobacter sp. MCI1899116. Consensus sequence marked with black color and non-consensus marked with white.


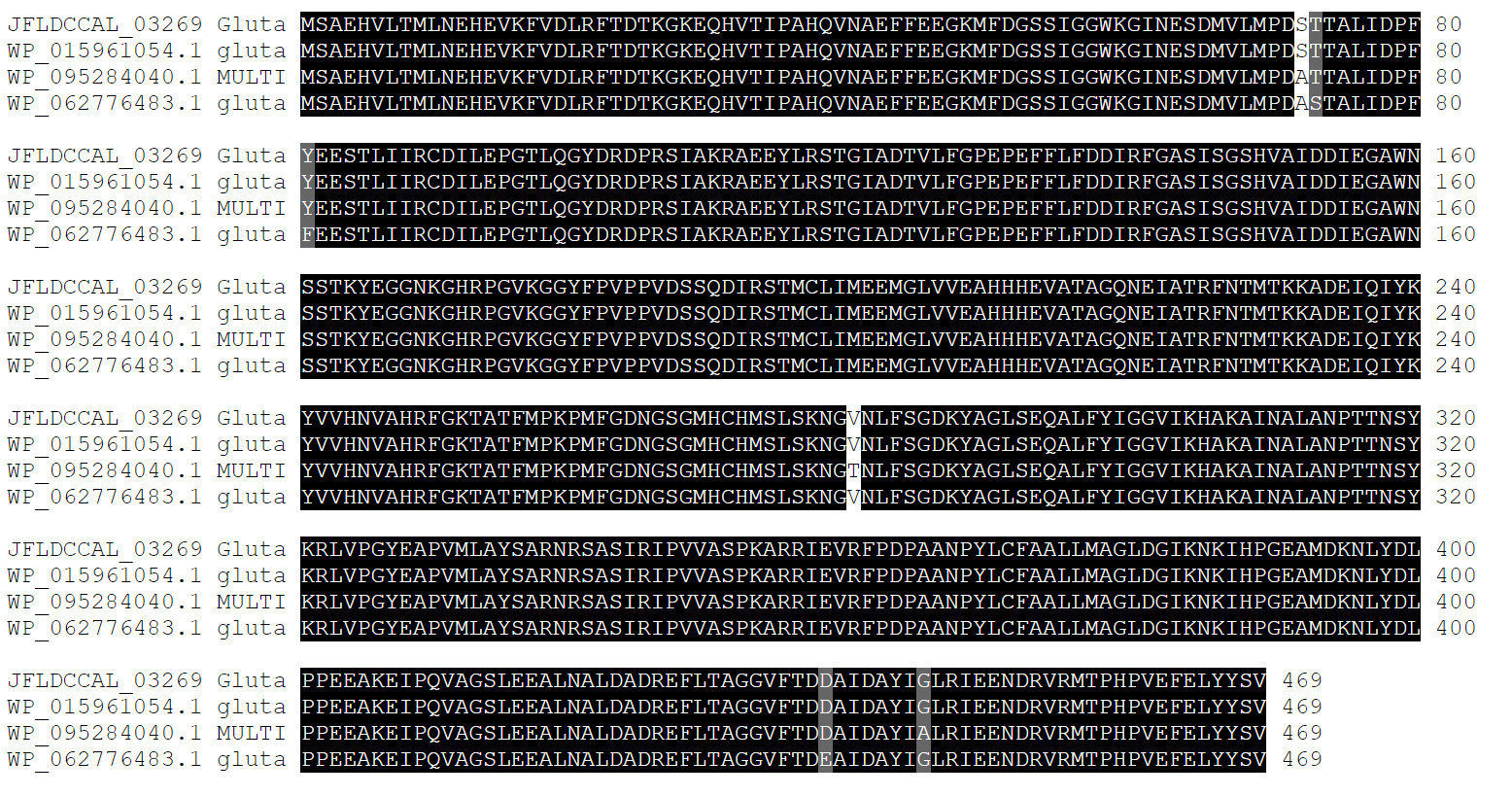


Figure S8: Multiple sequence alignment of glutamine synthetase I of Lelliottia amnigena PTJIIT1005 (JAKRZN000000000) similar to Lelliottia amnigena WP_015961054, Lelliottia WP_095284040, and Kluyvera intermedia WP_062776483. Consensus sequence marked with black color and non-consensus marked with white.


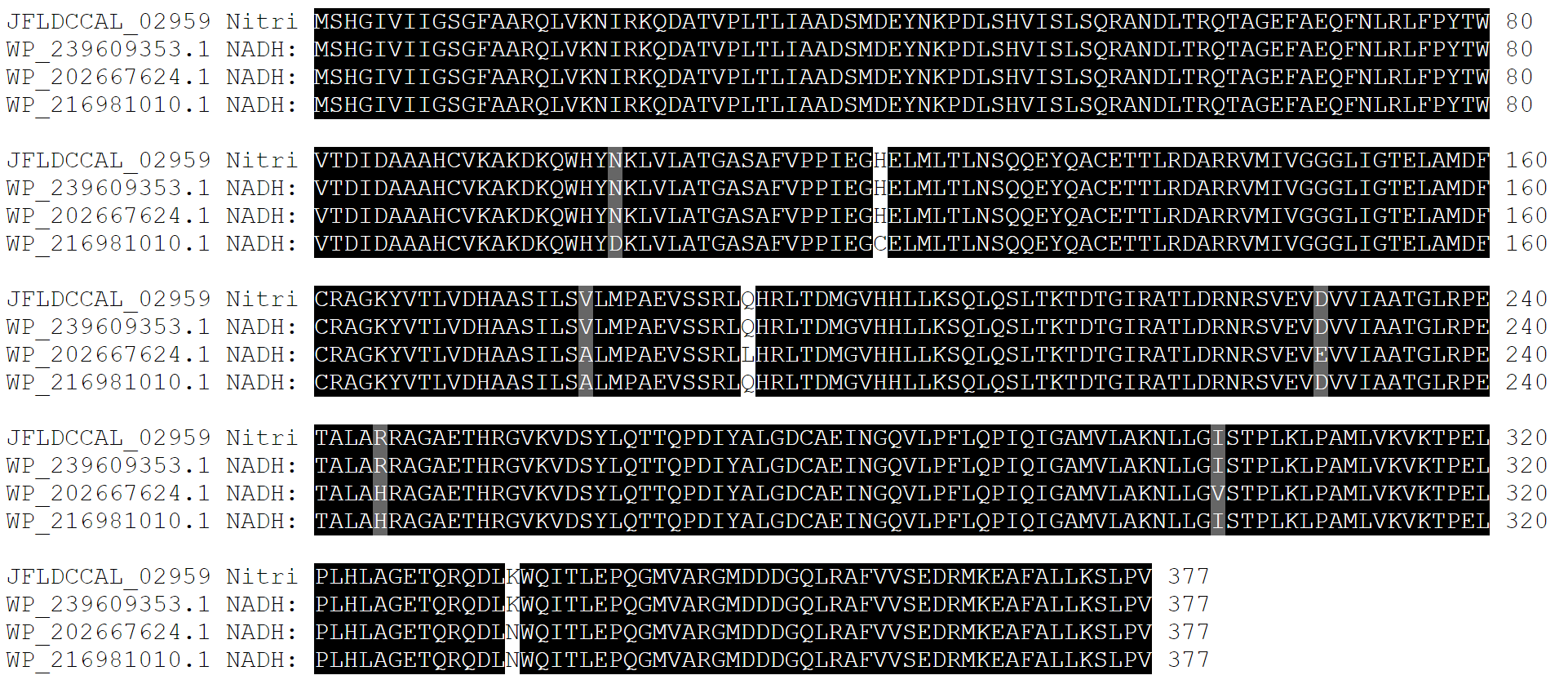


Figure S9: Multiple sequence alignment of nitric oxide reductase of Lelliottia amnigena PTJIIT1005 (JAKRZN000000000) similar to Lelliottia amnigena WP_239609353, Lelliottia WP_202667624, and L. amnigena WP_216981010. Consensus sequence marked with black color and non-consensus marked with white.


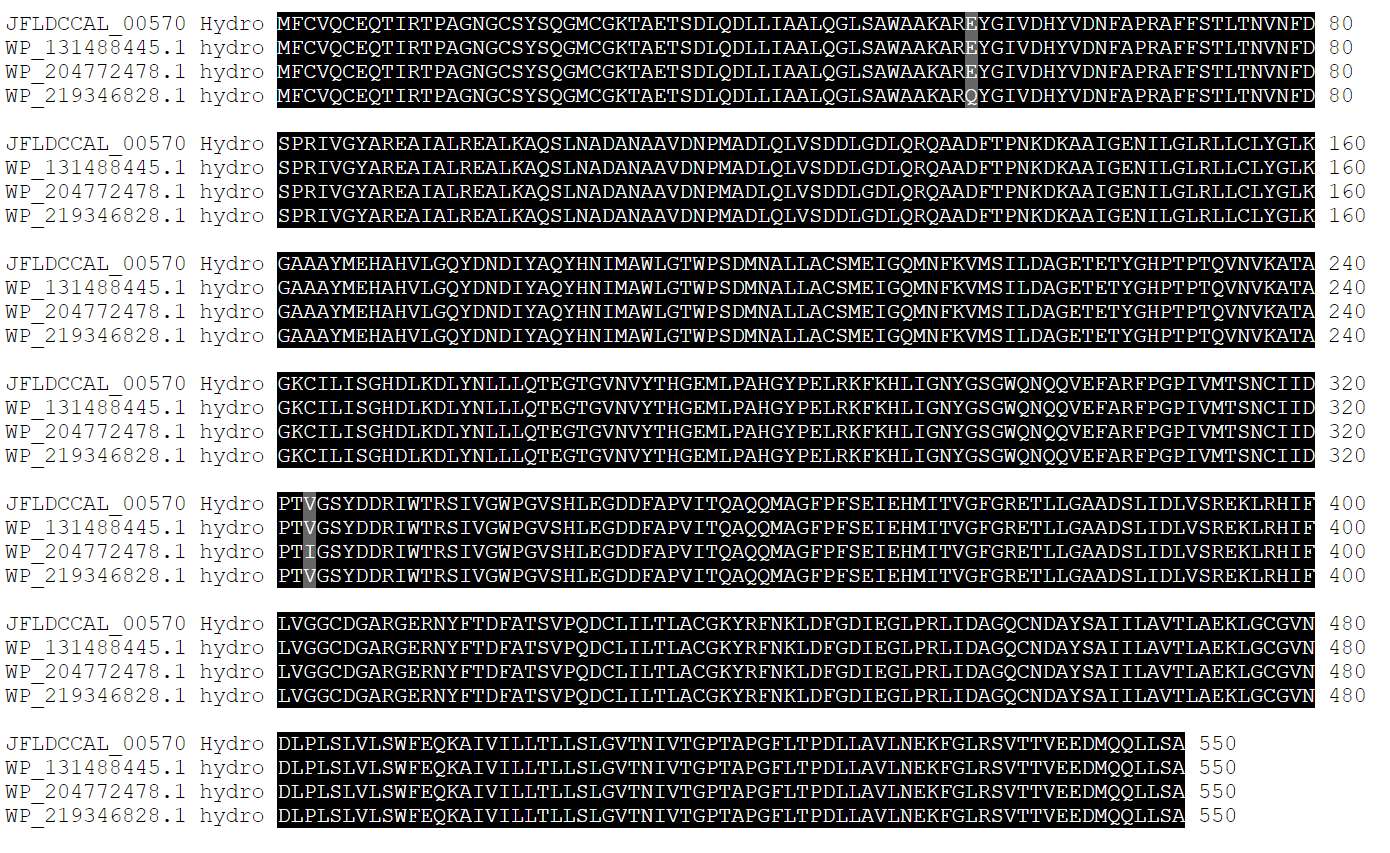


Figure S10: Multiple sequence alignment of hydroxylamine reductase of Lelliottia amnigena PTJIIT1005 (JAKRZN000000000) similar to Lelliottia amnigena WP_131488445, Lelliottia WP_204772478, and Lelliottia WP_219346828. Consensus sequence marked with black color and non-consensus marked with white.
